# Diagnosis and treatment of invasive pulmonary aspergillosis in critically ill intensive care patients: executive summary of the German national guideline (AWMF 113-005)

**DOI:** 10.1007/s15010-025-02572-2

**Published:** 2025-06-04

**Authors:** Dominic Wichmann, Martin Hoenigl, Philipp Koehler, Christina Koenig, Frederike Lund, Sebastian Mang, Richard Strauß, Markus A. Weigand, Christian Hohmann, Oliver Kurzai, Claus Heußel, Matthias Kochanek

**Affiliations:** 1 Department of Intensive Care Medicine, University Medical Centre Hamburg-Eppendorf, Hamburg, Germany; 2 Division of Infectious Diseases, Department of Internal Medicine, Medical University of Graz, Graz, Austria; 3 Translational Mycology, ECMM Excellence Centre, Medical University of Graz, Graz, Austria; 4 Faculty of Medicine, Department I of Internal Medicine, University of Cologne, and University Hospital Cologne, Cologne, Germany; 5 Centre for Integrated Oncology Aachen Bonn Cologne Duesseldorf (CIO ABCD), Division for Clinical Immunology, Cologne, Germany; 6 Department of Anaesthesiology, University Hospital Heidelberg, Heidelberg, Germany; 7 Department of Medicine 1, University Hospital Erlangen, Erlangen, Germany; 8 Department I of Internal Medicine, Department of Intensive Care Medicine, Klinikum Bremen-Mitte, Bremen, Germany; 9 Department of Intensive Care Medicine, Agaplesion Diakonieklinikum Rotenburg, Rotenburg (Wuemme), Germany; 10 Institute for Hygiene and Microbiology, Julius-Maximilians-University Würzburg, Würzburg, Germany; 11 National Reference Centre for Invasive Fungal Infections NRZMyk, Leibniz Institute for Natural Product Research and Infection Biology, Hans Knöll Institute, Jena, Germany; 12 Diagnostic and Interventional Radiology, University Hospital Heidelberg, Heidelberg, Germany; 13 Diagnostic and Interventional Radiology with Nuclear Medicine, Thoraxklinik at University Hospital Heidelberg, Heidelberg, Germany; 14 Translational Lung Research Centre (TLRC) Heidelberg, German Center for Lung Research (DZL), Heidelberg, Germany

**Keywords:** Invasive pulmonary aspergillosis, Intensive care medicine, Critically ill patients, Guideline, Azoles, Drug interactions

## Abstract

**Purpose:**

The executive summary of the guideline aims to provide the most relevant recommendations on the diagnosis and treatment of invasive pulmonary aspergillosis in critically ill patients in the intensive care unit.

**Methods:**

The guideline’s work included a systematic literature search, selection and assessment of the data relevant to the issues identified. Key questions included the areas of epidemiology, risk factors, diagnostics, and therapy. They were discussed analogous to a PICO scheme within the guideline committee, with subsequent working groups proposing recommendations for specific key questions, which were then again discussed and finalized by the entire guideline committee.

**Results:**

In addition to the classic risk factors (persistent neutropenia, allogeneic stem cell transplantation, congenital or acquired immunodeficiency, etc.), decompensated liver cirrhosis, COPD, solid tumours and viral pneumonia (influenza, COVID-19) have been established as risk factors for critically ill patients in need of intensive care. If there is no adequate improvement or even further clinical deterioration of the respiratory status in critically ill patients, the presence of IPA should be considered and appropriate diagnostic tests should be initiated. Diagnostics should include a CT scan of the chest and a broncho-alveolar lavage with culture for moulds, testing for galactomannan and PCR. Isavuconazole and voriconazole are recommended as first-line treatment, liposomal amphotericin B as an alternative, with posaconazole (PCZ) or the echinocandins (as an add-on to azole or polyene treatment) being additional options for salvage treatment.

**Conclusion:**

Invasive aspergillosis in critically ill patients represents a diagnostic and therapeutic challenge. If indicated, invasive aspergillosis should be considered and appropriate diagnostic tests initiated. Isavuconazole and voriconazole are recommended as first-line treatment, liposomal amphotericin B as an alternative.

**Supplementary Information:**

The online version contains supplementary material available at 10.1007/s15010-025-02572-2.

## Background

With the criteria for invasive fungal infections in intensive care patients published recently, a broad consensus on the (research) definition of invasive aspergillosis in critically ill patients in the intensive care unit (ICU) was established [1]. In order to create robust evidence, the guideline, which is primarily intended to harmonise research questions followed a conservative approach and recommendations are therefore very strict. In a clinical setting, deviations from these recommendations may be warranted and should be made in exceptional cases, when justified.

### Methods

The S1 guideline aimed to provide a comprehensive overview of evidence-based recommendations on the diagnosis and treatment of invasive (pulmonary) aspergillosis and addresses physicians involved in the care of critically ill adult patients treated in the ICU. This compendium highlights the key diagnostic and treatment recommendations from the guideline that are most important for clinical practice.

### Critical evaluation of evidence and Preparation of recommendations

The guideline presented is based on a systematic search, selection, and assessment of the data relevant to the identified issues. Due to the significant lack of randomised controlled trials (RCT) or comparable studies, it was generally not possible to assign recommendation grades or determine the quality of evidence. Therefore, as defined by the *Arbeitsgemeinschaft wissenschaftlicher Fachgesellschaften* (AWMF), the recommendations presented here are classified as expert opinions (S1 level).

## Determination of guideline questions and Preparation of the recommendations

Key questions were formulated for the areas of epidemiology and risk factors as well as diagnostics and therapy. Relevant core questions were identified and discussed within the guideline committee analogous to a PICO-based scheme. Subsequently, working groups for identified key questions were set up, who performed a literature search and formulated specific recommendations, which then underwent a review process within the entire guideline group. The final manuscript was submitted to the boards of the scientific societies and approved for publication.

### Systematic literature research

We used MEDLINE, Livivo and ScienceDirect for the literature search. Articles published before August 31st 2024 were taken into account. Search strings included: (“ICU OR intensive care OR critical care”) AND “aspergill* AND (“galactomannan OR aspergillus antigen OR LFA OR lateral flow OR glucan OR BDG”), (“Aspergill*) AND (invasive OR infection OR case OR patient OR report) AND (guideline OR treatment OR therapy OR diagnosis OR therapeutic drug monitoring”), (Aspergill*) AND (invasive OR infection) AND (ICU OR critical care OR critical illness OR intensive care) AND (risk OR factor OR epidemiology OR incidence OR mortality).

### Key recommendations

### Epidemiology

Invasive mould infections are predominantly caused by *Aspergillus* spp. The majority of infections (90%) are due to species from the *A. fumigatus* species complex, followed by species from the complexes *A. flavus*, *A. niger*, *A. terreus* [2–4]. The incidence of invasive pulmonary aspergillosis (IPA) varies depending on the patient group, geographical location, and also because, until recently [1], there was no consensus on the underlying diagnostic criteria. In addition to differentiate between colonisation and infection might be difficult when indirect methods such as antigen- or nucleic acid amplification tests are used and histology is not available [3]. The incidence of invasive pulmonary aspergillosis in critically ill patients in intensive care units is very likely underestimated, as suggested by results from retrospective autopsy studies, in which 2.8% had invasive aspergillosis, but only 40% of those cases had been identified ante mortem [5]. Significant differences also exist in the data on patient mortality, as IPA usually occurs in patients with pronounced disease severity and already significantly increased mortality risk [6]. In a retrospective cohort study with 1850 included patients in a medical intensive care unit in Leuven, Belgium, 6.9% of patients had microbiological or histopathological evidence of infection with *Aspergillus* spp. In this study the proportion of confirmed or probable IPA in patients without haematological cancer was 3.7%, in these the mortality rate was 90% [4].

### Risk factors for invasive aspergillosis

In recent years, additional risk factors for IPA in critically ill patients [6] have been identified. In addition to the classic risk factors [7], like (prolonged) neutropenia (< 500/mm³), post allogeneic stem cell transplantation or presence of haematological or solid cancer, post organ transplantation and patients with congenital or acquired immunodeficiency (CGD, AIDS, HIV infection with neutropenia) [6, 7], these include, COPD [8], ARDS [9], mechanical ventilation, viral infections from influenza and SARS-CoV-2 [10, 11], liver cirrhosis [12, 13], prolonged steroid therapy. Table 1 provides an overview of the risk factors for invasive aspergillosis described in the literature. In the past, a number of algorithms and diagnostic criteria for IPA have been proposed. To harmonise further studies, recently an international consensus definition for IPA has been published [1].

### Diagnostics

### Indication

**Fig. 1:**
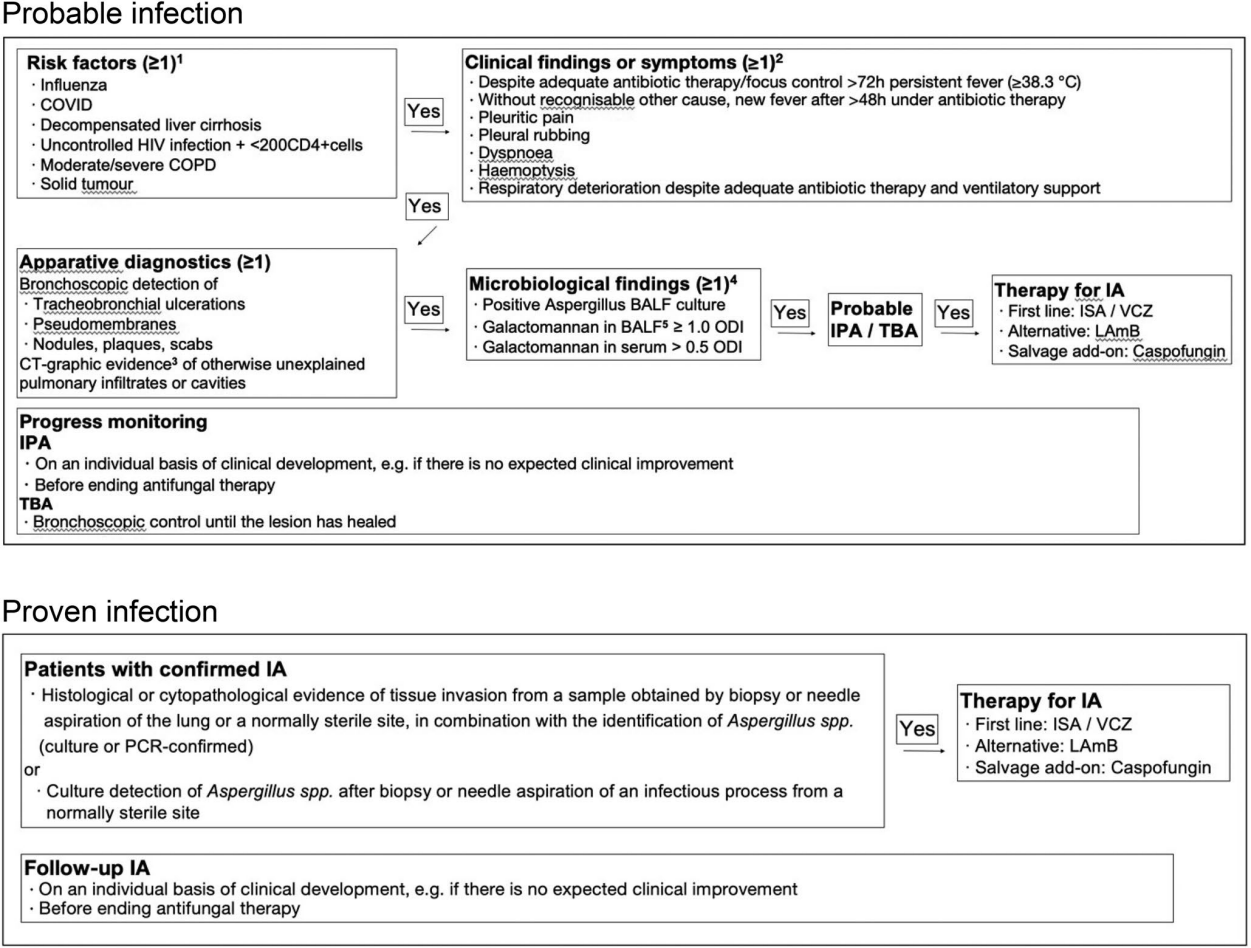
Diagnostic and therapeutic algorithm for invasive (pulmonary) aspergillosis. ^1^ Classic risk factors (e.g. allogeneic stem cell transplantation) are covered by the established EORTC/MSG definition [7]. ^2^ oligosymptomatic course possible, especially in isolated TBA. ^3^ not in isolated TBA. ^4^ in isolated TBA, galactomannan and culture from bronchoalveolar lavage may be negative, in which case diagnosis from tracheal aspiration/bronchial lavage is recommended. ^5^ BALF highly preferred over serum. IPA invasive aspergillosis, BALF bronchoalveolar lavage fluid, COPD chronic obstructive pulmonary disease, COVID-19 coronavirus disease 2019, CT computed tomography, HIV human immunodeficiency virus, ICU intensive care unit, IPA invasive pulmonary aspergillosis, ODI optical density index, TBA tracheobronchial aspergillosis, ISA isavuconazole, VCZ voriconazole, LAmB liposomal amphotericin B. Due to the lack of standardization, the consensus recommendation [1] does not yet generally favor NAT for diagnostics. However, in experienced centers, it can be helpful in identifying species and resistance markers.

With the publication of the criteria for invasive fungal infections in ICU patients at the end of March 2024, a broad consensus on the (research) definition of IPA in critically ill patients in the ICU has been established [1]. In order to create robust evidence, the guideline, which is primarily intended to harmonise research questions, is very conservative and the recommendations are therefore very strict. In a clinical setting, deviations from these recommendations may be appropriate and should be made in exceptional cases, when justified. Taking these aspects into account, our proposed diagnostic (and therapeutic) algorithm is shown in Fig. 1.

### Radiological imaging

Recommendations for the radiological diagnosis of fungal diseases were recently published [14, 15]. Radiological imaging provides a major contribution to the differential diagnosis of the causes of respiratory deterioration in intensive care patients. Bedside techniques like ultrasound or supine chest radiography do not provide sufficient information on early detection, characterisation, or monitoring of fungal infections. The following techniques are recommended for suspected IPA with or without extrapulmonary manifestations. If haematogenous spread is suspected, further imaging of the extra-thoracic regions may be necessary, for which recommendations are given below.

Lungs:

Native thin-slice CT, if possible, using low-dose technology. An angio-CT to investigate possible vascular invasion is recommended in case of haemoptysis. The characterisation of the infiltrates allows for a certain classification of the underlying causes, yet cannot replace microbiological diagnostics. Monitoring with low-dose CT is recommended if new symptoms develop, a failure to achieve the expected improvement or a deterioration of the clinical status is observed.

Brain:

The recommended examination modality is MRI with contrast medium. CT should only be performed in an acute situation, in particular to search for haemorrhage. If the CT remains unremarkable, MRI should be added, if the clinical situation allows it and no contraindications exist.

Paranasal sinuses:

Invasive aspergillosis of the paranasal sinuses may be oligosymptomatic, and clinical examination in intensive care patients is difficult. Therefore, either CT or MRI should be performed when in doubt. The MRI however, is more sensitive for visualisation of invasion of fungal mass into the orbits or brain. Furthermore, repeated CT scans of the eye can trigger cataract particularly in young people [16, 17].

Abdomen:

In ICU patients, the potentially greater insight gained from MRI of the abdomen is limited by the impaired adherence to repeated breath-holding. Therefore, multiphasic contrast-enhanced CT is recommended for ICU patients.

Transcutaneous CT-guided biopsy.

In view of the critical condition of ICU patients, a biopsy should be performed after careful risk-benefit assessment. Nonetheless, transcutaneous CT-guided biopsy is a reliable method for confirming the diagnosis, which might be preferable to empirical therapy if long-term therapy and possible secondary prophylaxis are required.

Follow-up.

It is recommended to repeat the imaging to monitor the response to therapy in the further course on an individual basis of the clinical development [16–19]. Especially before stopping therapy, a follow-up imaging is recommended.

### Bronchoscopy

The list of differential diagnosis to pathogen-induced deterioration of lung function in ICU patients is extensive (secretions, pulmonary oedema, bleeding, etc.). In addition to its diagnostic role, bronchoscopy is often also of therapeutic importance in the ICU setting. The risk of respiratory deterioration during a bronchoscopy is generally low [20], which is why invasive diagnostics should be performed at a low threshold, especially in intubated patients. In the case of tracheobronchial aspergillosis, bronchoscopy offers the advantage of direct visualisation and the possibility of targeted biopsies. Microbiological cultures, PCR and serological tests should be carried out on the broncho-alveolar lavage fluid (BALF) obtained in regions suspected of being IPA in CT.

### Culture

A positive BALF culture for *Aspergillus* spp. is associated with IPA in up to 50% of patients in the ICU [21], in addition to this, culture allows for species identification and phenotypic resistance testing to identify azole resistant isolates. However, culture alone has a low sensitivity of 20–50% [22] and can not differentiate between colonisation and infection.

### Serology

ELISA based testing for galactomannan from serum demonstrated a reasonable test performance in neutropenic patients [23]. In contrast to this in non-neutropenic patients the infection is usually not angio-invasive, and the test has a much lower sensitivity [24, 25]. This explains why galactomannan testing in non-neutropenic ICU patients should only be performed from respiratory materials [26]. Using a 1.0 ODI cut-off a multicentre study in ICU patients could demonstrate a sensitivity of 80% and a specificity of 97% [27]. Lateral flow assays for bedside testing have also been evaluated in ICU with similar performance measures [28].

The testing of beta-D-glucan (BDG) in the BALF as a panfungal biomarker for the diagnosis of IPA in the intensive care unit is not recommended, due to low specificity and false positive test results [29, 30].

### Nucleic acid amplification technique (NAT)

Molecular detection methods for the detection of *Aspergillus* spp. have been described from blood (also plasma, serum) and from deep airway materials, but the evidence for the benefit in critically ill patients in intensive care units is currently insufficient [1]. In addition, NAT does not differentiate between colonisation and infection. However, in combination with sequencing, it can be used for the targeted detection of azole resistance-associated mutations (RAMs) in the cyp51A gene. Studies on NAT from BALF have shown limited sensitivity and specificity, even in high-risk populations. However, this increased significantly when the PCR was combined with detection of the vitality of the fungal spores (galactomannan test) [31, 32]. In a systematic review regarding immunocompromised patients, molecular detection methods from blood showed a sensitivity and specificity of 79.2% and of 79.6%, respectively, for a single positive test result and 59.6% and 95.1% for two consecutive positive test results [33].

The Supplement Table 1 provides an overview of the various microbiological tests, their strengths and weaknesses.

**Table 1:** Risk factors for invasive (pulmonary) aspergillosis

Risk factors for IPA		Literature
ICU specific criteria*	Respiratory viral infection - Influenza - COVID-19	[1, 11, 70, 71]
	AIDS, HIV infection with neutropenia	[1, 9, 71–73]
	Liver disease, especially advanced liver cirrhosis, acute on chronic liver failure	[1, 6, 13]
	Solid tumour	[1, 73]
	COPD	[1, 4, 6, 74]
(Prolonged) neutropenia (< 500/mm³)		[7, 19]
Haematological malignancy		[7, 12, 19]
Allogeneic stem cell transplantation		[7, 12, 19]
Prolonged treatment with corticosteroids **		[7, 75]
Other immunosuppressive drugs - T-cell immunosuppressants, e.g. calcineurin inhibitors, TNF-alpha inhibitors, lymphocyte-specific monoclonal antibodies: Calcineurin inhibitors, TNF-alpha inhibitors, lymphocyte-specific monoclonal antibodies, immunosuppressive nucleoside analogues - B-cell immunosuppressants, e.g. ibrutinib		[7, 19]
Acute and chronic graft-versus-host reaction after allogeneic stem cell transplantation		[7, 19]
Solid organ transplantation, especially lung transplantation		[7, 75–74]
Congenital immunodeficiency		[7]
ARDS		[6]
Smoking		[6]
Alcohol abuse		[6]
Colonisation with *Aspergillus* spp.		[78, 79]
Environmental exposure to moulds (construction activity, plant soil, food)		[19]

* Established for research harmonisation [1]

** With steroids, recent data suggest that, in addition to the dose and duration of treatment, there are also differences between individual steroids in terms of the risk of invasive aspergillosis [80]

### Treatment

### Systemic therapy

The recommended first-line treatment for possible, probable and proven invasive pulmonary aspergillosis is either voriconazole (VCZ) or isavuconazole (ISA) intravenously [19, 34]. The reason for this is the landmark study for VCZ from 2002, which established VCZ as the standard medication [35]. However, VCZ can have disadvantages in patients treated in ICU, so that the use of ISA, as the alternative first-line treatment option, may be justified. With inhibition of various cytochrome P450 (CYP) enzymes, such as CYP2C19, CYP2C9 and CYP3A4, VCZ is a drug most frequently associated with major drug-drug interactions (DDI) in the ICU [36]. The main side effect of both, VCZ and ISA, is an increase in transaminases. Phototoxicity and neurological side effects as well as QTc prolongation have also been described with VCZ, whereas QTc shortening is reported for ISA. Limited data are available for ISA for intensive care use outside the treatment of haematology patients, however ISA has a more favourable pharmacokinetic profile compared to VCZ and is associated with fewer toxicities [33]. Therefore, ISA can be considered an attractive alternative first-line treatment; however, it is important to remember that ISA itself is metabolised via CYP3A4 and is therefore not entirely free from DDIs, although those are generally less pronounced compared to VCZ.

Liposomal amphotericin B (LAmB) is an alternative option for the treatment of IPA in the intensive care unit [17, 19]. However, LAmB is nephrotoxic and can therefore lead to a deterioration in renal function (which is usually reversible), particularly in patients who already have acute kidney injury. LAmB should also be used empirically as initial therapy in cases of suspected azole resistance due to local epidemiology and considered when relevant DDIs with azoles are expected [37]. Possible alternative second-line options could be posaconazole (PCZ) or the echinocandins. Echinocandins should not be used as first-line monotherapy. However, if no other options are available, they can also be used as add-on salvage therapy in combination with azole antifungals [38]. Our proposed diagnostic (and therapeutic) algorithm is shown in Fig. 1.

### Inhalation therapy

Inhalation of antifungal drugs is generally not recommended. It may be used off-label in individual cases but is technically very complex, especially in intubated patients. Best evidence exists for lung transplant patients with infection of the anastomosis [39]. For details, we refer to the information on antimicrobial inhalation therapy from the German Respiratory Society [40].

### Surgical therapy

With the exception of rare individual cases, the use of surgical therapy is limited in the intensive care setting.

### Drug-Drug interactions

Due to the inevitable polypharmacy in the intensive care setting, DDIs are frequent and affect approximately 30% of patients treated with mould-active antifungals [41].

These interactions involve alterations in metabolism and clearance, most frequently due to CYP-inhibition by azoles; resulting in risks for undesirable side effects, mitigation and amplification of other co-prescribed agents. However, in intensive care medicine, an individualised and interdisciplinary assessment of DDI is essential. This evaluation should consider clinical context, available monitoring options, potential risks and the efficacy of alternative therapies [42]. The use of DDI databases [42] as screening tools, in particular for azole therapies, is beneficial as they offer general recommendations on dosage adjustments and DDI management [43]. Potential DDI and their effects on the metabolism of agents commonly prescribed in the ICU setting are summarized in Table 2. However, these should not be regarded as comprehensive and can only serve as a guiding reference.

**Table 2:** Extract of important drug-drug interactions for mould-active Azole agents

Drug substance Class	Drug substance examples	Interaction
Anti-infectives	Clarithromycin	↓ Metabolism, ↑ Exposure and ↑ Effect of the macrolide (e.g. QTc prolongation)
	Rifampicin, rifabutin	↑ Azole metabolism, ↓ Azole exposure and ↓ Efficacy of azole
Chemotherapeutics	Cyclophosphamide, ifosfamide, protein kinase inhibitors	↓ Metabolism/efflux, ↑ Effects of chemotherapeutic agent (e.g. toxicity)
Immunosuppressants	Calcineurin/mTOR inhibitors	↓ Metabolism/efflux, ↑ Exposure and ↑ Effects of immunosuppressants
Sedatives	Benzodiazepines	↓ Metabolism, ↑ Exposure and ↑ Effects of benzodiazepine
Analgesics	Opioids	↓ Metabolism, ↑ Exposure and ↑ Effects of opioid
Anticonvulsants	Phenytoin, carbamazepine	↑ Azole metabolism, ↓ Azole exposure and ↓ Efficacy of azole
Neuroleptics	Quetiapine, haloperidol	↓ Metabolism, ↑ Exposure and ↑ Effects of neuroleptics (e.g. QTc prolongation)
Cardiac	Amiodarone, Ca^2+^ channel blockers, digoxin, ivabradine	↓ Metabolism, ↑ Exposure and ↑ Effects of cardiac drugs (e.g. QTc prolongation)
HIV therapeutics	Ritonavir, efavirenz	Complex interaction (inhibitor/substrate), possibly ↓ Efficacy of the azole
Oral anticoagulants	Oral anti-Xa inhibitors, phenprocoumon	↓ Metabolism, ↑ Exposure and ↑ Effects of anticoagulants

### Therapeutic drug monitoring (TDM)

### Voriconazole

VCZ shows pronounced intra- and inter-individual pharmacokinetic variability due to its non-linear elimination. In addition to DDI, polymorphisms of the CYP2C19 enzyme can also contribute to fluctuations in VCZ concentrations [44]. Several studies have demonstrated a correlation between VCZ serum concentration and efficacy. Thus, based on systematic reviews and a recent meta-analysis, a therapeutic target range of 1-5.5 mg/l is currently being recommended [45]. For patients with severe infections (e.g. multifocal or disseminated), central nervous system (CNS) involvement, and infections with pathogens with an elevated MIC, an increased target range of 2–6 mg/l is recommended [34, 46–48]. Supra-therapeutic VCZ concentrations have been associated with neuro- and hepatotoxicity [49], and a meta-analysis identified VCZ levels > 6 mg/l as strong predictors for toxicity [45]. VCZ concentrations should ideally be monitored within the first 5 days of therapy, with repeats four days after dose adjustments.

### Isavuconazole

ISA TDM is currently not recommended in routine practice due to its dose-proportional pharmacokinetics, moderate inter-patient variability, and lack of defined efficacy or toxicity thresholds [50]. In the SECURE trial, fewer than 3% of patients had ISA levels outside the 1–7 mg/L range [51] and Desai et al. found no significant link between ISA levels and mortality or treatment response. However, TDM may be useful in specific ICU populations at risk for altered potentially sub-therapeutic drug exposure, such as those on renal replacement therapy, extracorporeal membrane oxygenation, or with high BMI [52, 53]. Due to its long half-life, TDM should be performed after 5–7 days. While > 1 mg/L is generally recommended (as > 90% of patients in SECURE achieved this), some experts suggest aiming for > 2 mg/L based on pharmacokinetic/dynamic models [54]. Toxicity has been shown to occur at levels > 4.8 mg/L [55, 56]. However, data from the SECURE trial and a study including intensive care patients, did not establish a clear correlation between ISA exposure and adverse reaction rates, highlighting the conflicting evidence [57].

### Posaconazole

In retrospective analyses of patients who received PCZ for prophylaxis or IPA therapy, breakthrough infections and a reduced response to therapy occurred more frequently in patients with low PCZ concentrations [58]. Furthermore, in a prospective study, PCZ TDM had a positive effect on the reduction of breakthrough infections [59].

For the treatment of IPA, achieving a mean PCZ concentration of 1.25 mg/l resulted in an improved response rate in patients receiving PCZ for salvage therapy [60]. In ICU patients, up to 35% of the measured PCZ concentrations were below the targeted range for IPA therapy [61]. Of note, PCZ concentrations in bronchoalveolar fluid can reach levels up to 40 times higher than in plasma [62]. This observation might explain why some studies failed to demonstrate a correlation between low PCZ plasma concentrations and treatment failure, as pulmonary drug penetration may still be sufficient despite low systemic levels. Yet, available guidelines currently recommend maintaining trough levels > 1 mg/l for the treatment of IPA [63]. To avoid toxicity, including hepatotoxicity and development of pseudo hypoaldosteronism, an upper limit of 2 or 4 mg/l is being discussed [51, 63, 64]. If TDM is performed, it is recommended on days 5–7 of therapy, although further monitoring may be required depending on the results and clinical context [63].

### Liposomal amphotericin B

Besides preclinical PK/PD-studies [65–67], clear data on target levels for LAmB in ICU patients with IPA do not exist and are further complicated by different LAmB kinetics in tissue und serum as well as accumulation in lung tissue [68, 69]. Therefore, TDM is currently not recommended.

### Echinocandins

Since most of the available data is based on animal models of candidiasis rather than IPA, TDM for echinocandins is not currently recommended.

## Conclusion

Invasive (pulmonary) aspergillosis in critically ill ICU patients presents a diagnostic and therapeutic challenge. If there is no improvement or if the respiratory status deteriorates further, the presence of IPA should be considered and appropriate diagnostic tests should be initiated. This should include a chest CT scan and a bronchoscopy with subsequent galactomannan testing and culture for moulds. In addition, TDM should be performed when appropriate for dose optimization.

## Electronic supplementary material

Below is the link to the electronic supplementary material.

Supplementary Material 1

Supplementary Material 2

## Data Availability

No datasets were generated or analysed during the current study.
