# Supplementary material for: Diagnosis and treatment of invasive pulmonary aspergillosis in critically ill intensive care patients: executive summary of the German national guideline (AWMF 113-005)
Source: Infection. 2025 Jun 4;53(4):1299–310. doi: 10.1007/s15010-025-02572-2 (PMC12316785; doi:10.1007/s15010-025-02572-2)
Supplement: Supplementary file 1 — Supplementary Material 1 [file 15010_2025_2572_MOESM1_ESM.docx]

**Table 5: Microbiological diagnostics for invasive aspergillosis**

| **Diagnostic test** | **Galactomannan** | **LFA** | **Beta-D-Glucan** | **PCR** | **Microscopy** | **Culture-based processes*** | **Histopathology** |
| --- | --- | --- | --- | --- | --- | --- | --- |
| **Material** | Serum,  BALF | Serum, BALF | Serum | Serum, sputum, cerebrospinal fluid, BALF | Blood, BALF, TA, sputum, cerebrospinal fluid, various others | Serum, sputum, cerebrospinal fluid, BALF | Tissue |
| **Early proof possible** | + | + | + | + | + | - | - |
| **Detection of a broad spectrum of pathogens** | - | - | ± | ± | ± | ± | - |
| **Identification down to species level** | - | - | - | + | - | ± | - |
| **Quantitative results** | + | +  (if Cube reader is used) | + | ± | - | - | - |
| **Commercial tests / staining** | Platelia™  Aspergillus; Euroimmun Aspergillus ELISA**, Virclia Assay**, and others | IMMY sõna Aspergillus GM LFA | Fungitell® Assay (FDA cleared) / Wako test | Fungiplex® Universal PCR | Calcofluor white with 10% potassium hydroxide or Grocott-Gomori Methenamine silver stain | Sabouraud dextrose agar and rarely brain-heart agar | Grocott-Gomori methenamine silver staining or periodic acid staining; immunohistology / FISH |
| **Sensitivity** | Serum 11-83%  BALF 58-95% | 91-97% | 55-96% | ~80% |  | 35-82% |  |
| **Specificity** | Serum 47-100%  BALF 87-100% | 92-98% | 77-96% | ~75% |  | 95-100% |  |
| **Fast turn around** | + | ++ | + | ± | ± | - | - |
| **Low costs** | ± (in house or shipping) | + | ± (in house or shipping | - | ± | ± | ± |
| **Comment** | False positive capabilities, best experience with hematology patients | False positive capabilities | False positive capabilities | Problem: Colonization vs infection | Investigator dependent; colonization vs infection | Problem: Colonization vs infection | Investigator dependent |

* Identification of the cultivated Aspergillus isolates using MALDI-TOF mass spectrometry, species-specific nucleic acid amplification methods or sequencing methods (detection or analysis of taxonomically applicable nucleotide sequences using specific PCR, gene sequencing or whole genome sequencing).

** are independently clinically validated
