# Supplementary material for: Diagnosis and treatment of invasive pulmonary aspergillosis in critically ill intensive care patients: executive summary of the German national guideline (AWMF 113-005)
Source: Infection. 2025 Jun 4;53(4):1299–310. doi: 10.1007/s15010-025-02572-2 (PMC12316785; doi:10.1007/s15010-025-02572-2)
Supplement: Supplementary file 2 — Supplementary Material 2 [file 15010_2025_2572_MOESM2_ESM.docx]

**Table 6: Treatment of invasive pulmonary aspergillosis and tracheobronchial aspergillosis [32, 81]**

| **Substance** | **Dosage** | **Dose adjustment** | | | **TDM target range**  **(mg/L)** | **Comment** |
| --- | --- | --- | --- | --- | --- | --- |
|  |  | **Renal insufficiency** | **Liver insufficiency** | **ECMO** |  |  |
| VCZ  [82] | D1: 2 × 6mg/kg/d i.v.  from D2: 2 × 4mg/kg/d i.v. | GFR <50ml/min based on risk-benefit assessment | Child A, B:  D1: 2x 6mg/kg  from D2: 2x 2mg/kg  Child C:  not specified | TDM recommended, especially after changing the oxygenator | 2 - 6 | First line  azole-sensitive strain |
| [76] | D1-2: 3 × 200 mg/d i.v.  from D3: 1 × 200 mg/d i.v. | none | Child A, B: none  Child C:  not specified | TDM recommended, especially after changing the oxygenator | 2 - 4.8 | First line  azole-sensitive strain |
| LAmB  [86] | 3mg/kg/d i.v. | none | unknown | unknown | unknown | Second line  Azole-resistant strain  Azole intolerance |
|  |  |  |  |  |  |  |
| [207] | D1: 2 × 300mg/d i.v.  from D2: 1 × 300mg/kg/d i.v. | GFR <50ml/min after risk-benefit assessment | none | TDM recommended, especially after changing the oxygenator | 1 - 3.5 | Possible alternative VCZ / ISA |
| Caspofungin  [77, 87, 208] | D1: 70mg/d i.v  from D2: 50mg/d i.v.; at KG >80kg further 70mg/d i.v. | none | Child C:  D1: 1x 70mg/d  D2: 1x 35mg/d | unknown | unknown | Add-on for VCZ / ISA  as salvage therapy  Add-on for VCZ / ISA as empirical therapy in settings with high levels of azole resistance |
| LamB inhalative  [209-212] | 50-100mg 2-3x/week | none | none | none | none | Not as monotherapy  Add-on e. g. after lung transplantation |
